# Non-invasive metabolomics biomarkers of production efficiency and beef carcass quality traits

**DOI:** 10.1038/s41598-021-04049-2

**Published:** 2022-01-07

**Authors:** Virginia M. Artegoitia, J. W. Newman, A. P. Foote, S. D. Shackelford, D. A. King, T. L. Wheeler, R. M. Lewis, H. C. Freetly

**Affiliations:** 1 USDA, ARS, Western Human Nutrition Research Center, 430 West Health Sciences Drive, Davis, CA 95616 USA; 2 USDA, ARS, Meat Animal Research Center, Clay Center, NE 68933 USA; 3 Animal Science, University Nebraska, Lincoln, NE 68583 USA; 4 Animal Science, Oklahoma State University, Stillwater, OK 74078 USA

**Keywords:** Metabolomics, Metabolomics, Metabolomics, Machine learning, Predictive markers, Metabolism, Animal biotechnology

## Abstract

The inter-cattle growth variations stem from the interaction of many metabolic processes making animal selection difficult. We hypothesized that growth could be predicted using metabolomics. Urinary biomarkers of cattle feed efficiency were explored using mass spectrometry-based untargeted and targeted metabolomics. Feed intake and weight-gain was measured in steers (n = 75) on forage-based growing rations (stage-1, 84 days) followed by high-concentrate finishing rations (stage-2, 84 days). Urine from days 0, 21, 42, 63, and 83 in each stage were analyzed from steers with the greater (n = 14) and least (n = 14) average-daily-gain (ADG) and comparable dry-matter-intake (DMI; within 0.32 SD of the mean). Steers were slaughtered after stage-2. Adjusted fat-thickness and carcass-yield-grade increased in greater-ADG-cattle selected in stage-1, but carcass traits did not differ between ADG-selected in stage-2. Overall 85 untargeted metabolites segregated greater- and least-ADG animals, with overlap across diets (both stages) and breed type, despite sampling time effects. Total 18-bile acids (BAs) and 5-steroids were quantified and associated with performance and carcass quality across ADG-classification depending on the stage. Stepwise logistic regression of urinary BA and steroids had > 90% accuracy identifying efficient-ADG-steers. Urine metabolomics provides new insight into the physiological mechanisms and potential biomarkers for feed efficiency.

## Introduction

Efficient and sustainable cattle farming requires producing quality beef while reducing operating costs and production wastes to meet demand from both producers and consumers in the complex U.S. beef production system^1^. The industry uses a wide range of nutritional inputs and animal types that span a diversity of geographies and climates. Cereal grains and grain by-products are the predominant dietary components during the finishing period while forage traditionally constitutes the main fraction in cattle feed during the growing period^2^. Breeding and nutrition programs during the last decades notably improved animal performance and carcass composition including cattle growth rates and fat deposition^3,4^. These types of economically relevant traits are controlled by many genes and environmental factors that are variably expressed contributing to the moderate prediction of high priority phenotypes selection for the beef industry^5^. Next-generation phenotyping using metabolomics is becoming a fundamental approach to refine trait description to improve the prediction of breeding values of the animals aligned with the objectives of selection programs^6–8^. This analytical technique provides a distinctive insight into the biochemical activity caused by environmental and genetic factors, and therefore can robustly correlate with complex phenotypes and identify biomarkers of specific physiological states^9^. A compiled composition of the bovine metabolome that sets the reference of metabolite values in the bovine tissues is already freely available^10^. Recent systematic reviews on livestock metabolomics applied in both research and industry summarized the importance of biomarker discovery for prognostic strategies in animal health/performance as well as increasing prediction accuracies^11^.

We previously identified biomarkers of the complex expression of average daily gain (ADG) in ruminal-fluid/plasma and multiple tissues by multivariate analysis during the finishing stage in steers^12–14^. These observations raise the possibility of using metabolomics for status monitoring of important production traits in beef cattle. Identifying metabolites of physiological expression for production efficiency and carcass quality may be a useful tool for cattle selection to improve sustainable beef production. Therefore, a non-invasive biofluid easy to collect overtime on a routine basis without the need for special training is crucial. In the current study, we aimed to examine urine comprehensive metabolomic profiling using untargeted/targeted LC–MS technology, with the goal of identifying urinary metabolite markers that can be used to predict feed efficiency and beef carcass quality traits on different breed types of steers on forage- and concentrate-based diets.

## Results

### Characteristics of steers in the selected ADG classification

In Table 1, the performance and carcass traits of selected ADG-classification is provided. The dry matter intake (DMI) did not differ between ADG groups in both stages; ADG was greater in the greater-ADG group (0.99 ± 0.08 kg/day; 2.25 ± 0.08 kg/day) than the least-ADG group (0.69 ± 0.05 kg/day; 1.90 ± 0.08 kg/day) for stage-1 and stage-2, respectively (*P* < 0.01). The feed conversion ratio was lower in the greater- ADG than least-ADG in both stages (*P* < 0.01). The residual feed intake (RFI) differed between ADG groups in stage-1 (*P* = 0.01) but not in stage-2. Final body weight of the steers did not differ (*P* > 0.29) within ADG-classification on 1/2-stages. Adjusted-fat-thickness and image analysis yield-grade improved in the greater-ADG in stage-1 (*P* < 0.05) while in stage-2 carcass traits did not differ (Table 1). There was no breed type effect on performance and carcass characteristics for ADG-groups for either stage (Supplementary Table S1). Steers classified as greater- or least-ADG in stage-1 were not the same steers classified in stage-2. The slope of ADG during concentrate feeding regressed on ADG during forage feeding did not differ from zero for the entire population (*P* = 0.89) nor did it differ for just the cattle selected in stage 2 (*P* = 0.18).

**Table 1:** Performance characteristics (means ± SE) of the steers selected at the end of stage-1 (forage) and stage-2 (concentrate) and carcass traits at slaughter according to differences in ADG with those with the greatest ADG and least ADG, whose dry matter intake was within 0.32 SD of the mean intake. Steers classified as greater- or least-ADG in stage-1 were not the same steers classified in stage-2. *Daily average dry matter intake (DMI); Average daily gain (ADG), Feed conversion ratio (FCR) Residual feed intake (RFI).

Variables	Stage 1			Stage 2		
	Least-ADG (n = 5)	Greater-ADG (n = 7)	*P*-value	Least-ADG (n = 8)	Greater-ADG (n = 8)	*P*-value
DMI (kg/day)	8.46 ± 0.14	8.62 ± 0.12	0.40	11.86 ± 0.11	12.11 ± 0.11	0.12
ADG (kg/day)	0.69 ± 0.03	0.99 ± 0.02	< 0.01	1.87 ± 0.04	2.24 ± 0.04	< 0.01
FCR	12.2 ± 0.3	8.7 ± 0.2	< 0.01	6.22 ± 0 .14	5.53 ± 0.13	< 0.01
RFI	0.26 ± 0.12	− 0.15 ± 0.10	0.02	− 0.10 ± 0.14	− 0.16 ± 0.14	0.77
Initial body weight (kg)	314 ± 10	303 ± 8	0.41	482 ± 9	438 ± 9	0.003
Final body weight (kg)	372 ± 11	386 ± 9	0.34	639 ± 9	635 ± 9	0.29
Age (day)	274 ± 1	270 ± 1	0.03	399 ± 1	396 ± 1	0.14
**Carcass traits (collected after stage-2)**						
Marbling score	535 ± 26	532 ± 31	0.95	488 ± 18	468 ± 17	0.43
Hot-carcass weight (kg)	395 ± 7.1	398 ± 6.9	0.77	405 ± 8.6	384 ± 8.1	0.10
Ribeye-area (cm)	13.3 ± 0.41	12.5 ± 0.42	0.22	13.9 ± 0.52	13.8 ± 0.50	0.86
Adjusted-fat-thickness (cm)	0.46 ± 0.05	0.62 ± 0.06	0.03	0.43 ± 0.04	0.47 ± 0.04	0.60
Yield grade	3.08 ± 0.15	3.79 ± 0.18	0.02	3.05 ± 0.26	2.71 ± 0.25	0.38
Fat thickness (cm)	0.42 ± 0.05	0.56 ± 0.05	0.12	4.43 ± 0.05	0.4 ± 0.04	0.87

### Urine metabolomic characterization of ADG-classification

**Figure 1:**
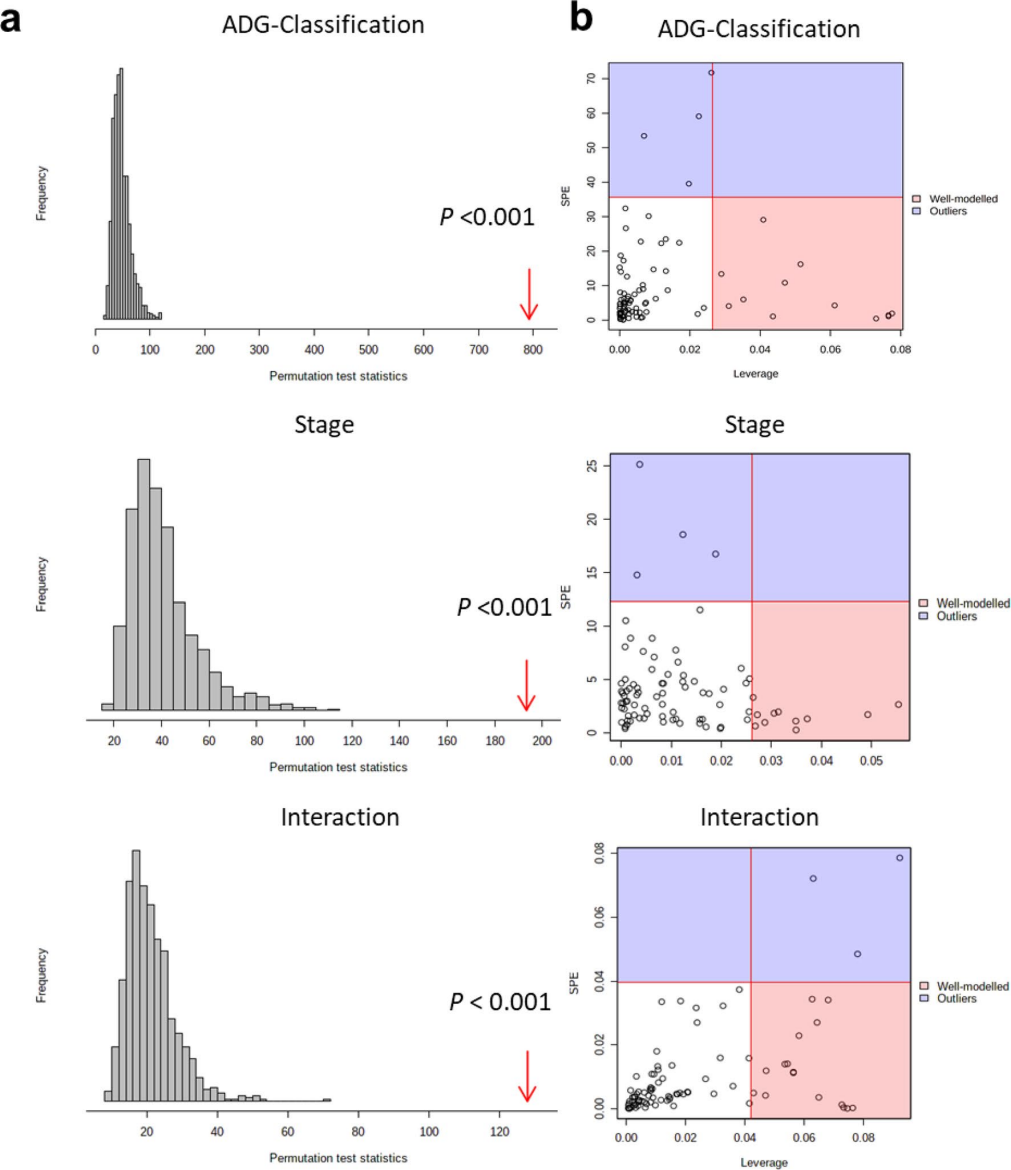
ANOVA-simultaneous component analysis (ASCA) for the ADG-classification, stage, and interaction. (**a**) Model validations through permutations, as demonstrated by significance levels of *P* < 0.01. (**b**) Leverage/SPE scatter plots of the ASCA variables submodels for ADG-classification, stage and their interactions. Metabolites in red have high loadings that follow the expression patterns of the submodels. Metabolites in blue have expression patterns that are different from the major patterns.

Overall, 85 metabolites were identified between the greater/least-ADG groups in both stages (1-forage diet; and 2-concentrate diet) and the six time samplings during growth (Supplementary Table S2). In order to analyze the overall metabolic shift over the entire timeframe of the study, ANOVA and ANOVA-simultaneous component analysis (ASCA) models were constructed with the resulting scores graphically displayed and grouped according to the time component of the study design. A scree plot was used to graphically display the relationship between eigenvalues and factors. The major patterns associated with factor A (time or stage variation), factor B (greater/least-ADG) and their interaction were shown in the score scatter plots (Supplementary Fig. S1) based on PC1 of the corresponding sub models. In a follow-up process, a significance test was performed by a permutation approach to validate the model for the ADG-classification, time or stage, and their interaction. Based on Supplementary Fig. S1, it is evident that although ADG-classification and time both have a dramatic impact (*P* < 0.01) on the urine metabolome, these changes were independent as indicated by no interaction (*P* > 0.8). However, stage and the interaction of stage by ADG-classification was significant (Fig. 1a). The significant variables associated with a specific factor were identified based on the leverage/squared prediction error (SPE) plots (Fig. 1b). A total of 12 metabolites were greatly altered by ADG-classification, 10 metabolites by stage including bile acids, steroids and fatty acids (Fig. 1b, Supplementary Table S3).

**Figure 2:**
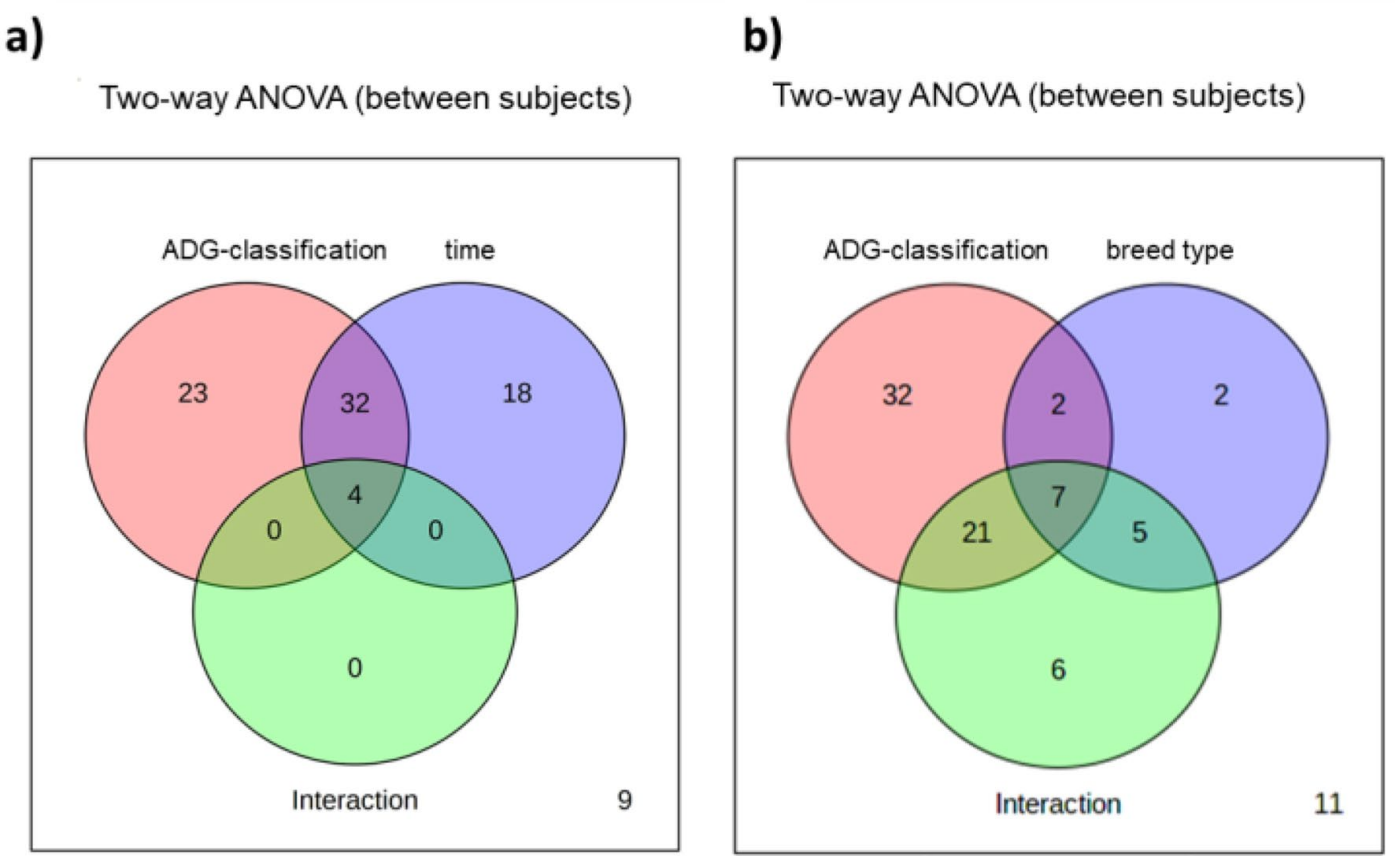
Two-way ANOVA analysis results showing different number of metabolites that have significant *P*-value (< 0.05) in time (**a**), breed type (**b**), ADG-classification, or the interaction of both variables.

**Figure 3:**
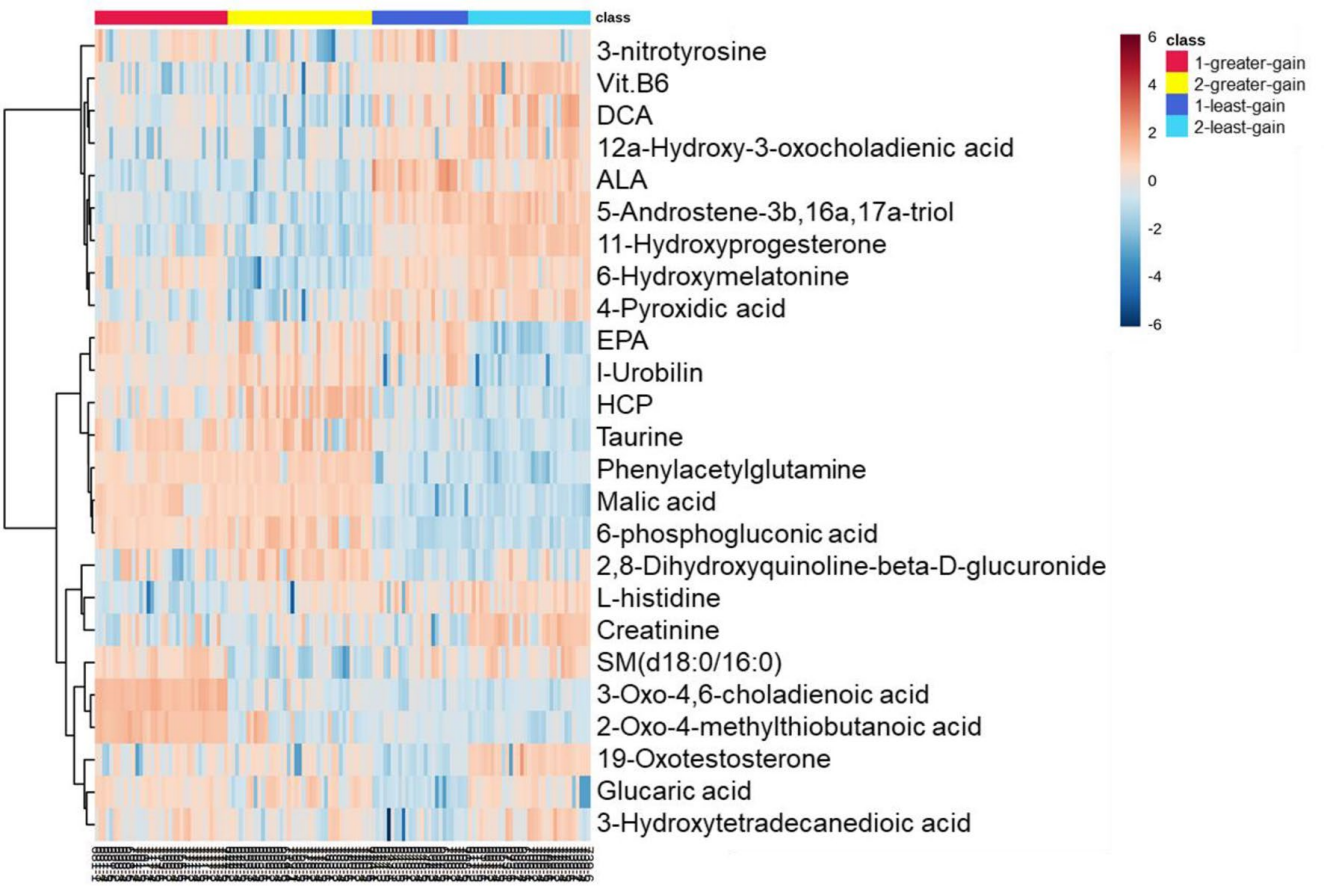
A heatmap representing the top 25 ANOVA metabolic changes in greater/least-ADG for stage 1 and stage-2 within 6 times point in stage 1 and stage 2. Dark-blue squares indicates a reduction of up to six folds, light-colors square indicates no significant fold change, and red square indicates an increase of up to six folds. *ALA* alpha-linoleic acid, *DCA* deoxicholic acid acid, *EPA* eicosapentaenoic acid, *HCP* heptacarboxyl porphyrin.

Considering time as an independent variable, a univariate two-way ANOVA analysis was performed to define the impact of ADG-classification for each stage, time, and their interactive effects. As shown in Fig. 2a, 59 metabolites showed a significant ADG-classification/stage effect, 54 metabolites showed a significant time effect, and 4 metabolites showed a significant interaction effect for each time point differing by ADG-classification (Supplementary Table S4). In total 59 metabolites differentially expressed between least/greater ADG were consistently expressed over time such that they could be considered as suitable model metabolites to reflect the ADG-specific phenotype. A heatmap (Fig. 3) was constructed to present the metabolic alterations of these identified metabolites of ADG-classification by stage (FDR < 0.05). Pathway enrichment analysis showed significant enrichment (*P*-values < 0.05, Supplementary Table S5) of several pathways in the ADG-classification, including steroid biosynthesis, primary bile acid metabolism, biosynthesis of unsaturated fatty acids alpha-linolenic acid metabolism, vitamin B6 metabolism, cysteine and methionine metabolism.

**Figure 4:**
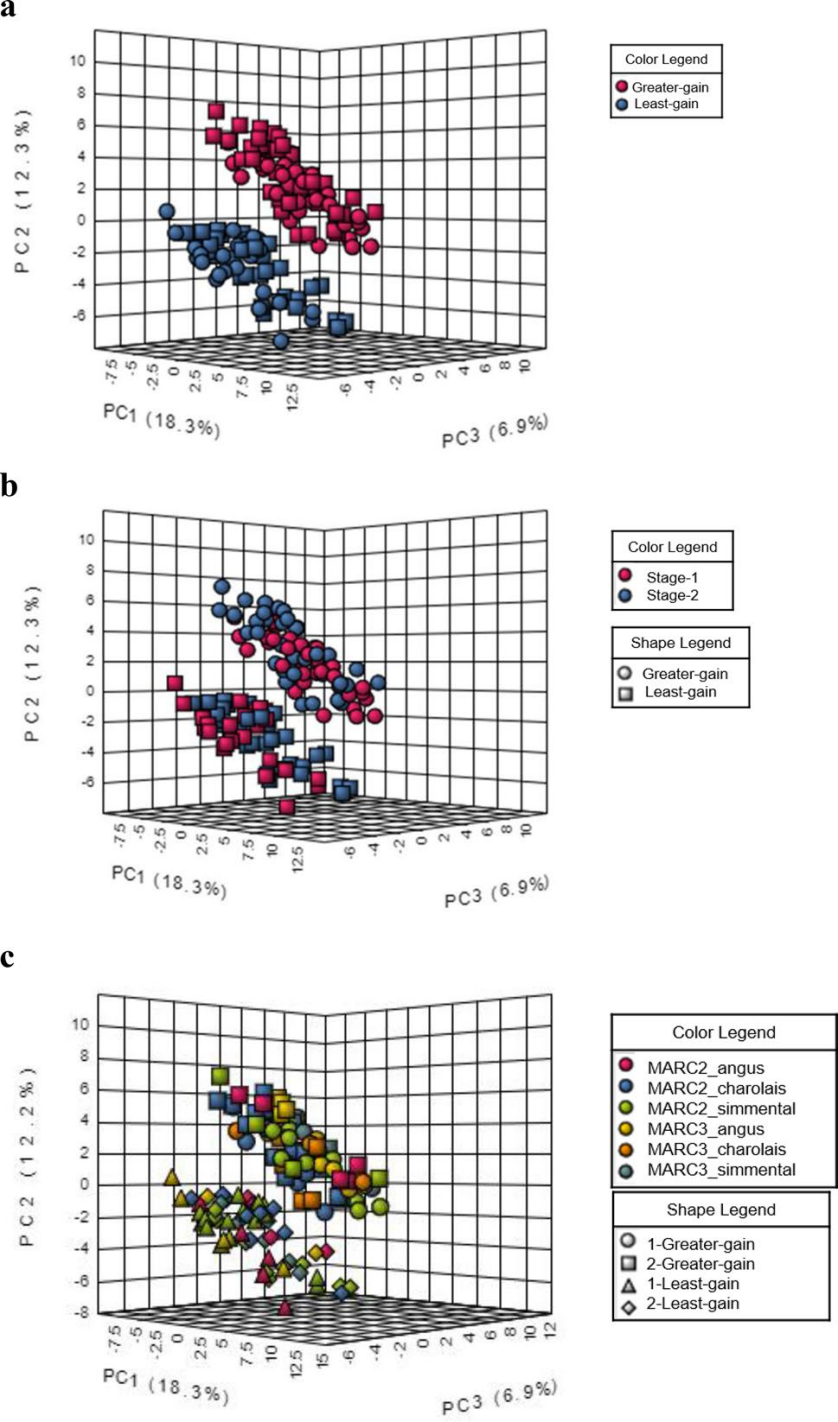
Interactive principal components analysis (iPCA) of untargeted metabolites of selected least-ADG vs. greater-ADG classification steers (**a**), and interactions with both stages (**b**) and, breed type (**c**).

The interactive principal components analysis (iPCA) represents the untargeted metabolites separation by ADG-classification and the interaction result with stage and breed type. (Fig. 4). There were 16 metabolites with identified breed type effects and 39 metabolites showing interaction of breed type that differentiated ADG-classification by each stage in the two-way ANOVA involved in bile acids, steroids, and protein metabolism (Fig. 2b; Supplementary Table S5). There were no identified untargeted metabolites in the MARC 3-Charolais cross in low-ADG phenotype in either stage (Fig. 4c). Therefore the urinary metabolome indicated that MARC3-Charolais cattle excrete lower levels of 11-oxo-androsterone glucuronide, 5-Taurinomethyl-2-thiouridine, 5-androstene-3b,16a,17a-trio, 12a-hydroxy-3-oxocholadienic acid, and higher levels of hepatic metabolites including cholesteryl acetate, bilirubin glucuronide and 24,25-Dihydroxyvitamin D3 than other breeds (Supplementary Fig. S2).

As a result of the ROC analysis of the untargeted metabolites, potential biomarkers were identified as androsterone sulfate, 11-hydroxyprogesterone-glucuronide deoxycholic acid glycine conjugated and taurochenodeoxycholate in stage-1 (AUC = 0.93, CI 0.83–0.99) while dexocholic acid glycine conjugated, progesterone and taurochenodeoxycholate were identified in stage-2 (AUC = 0.99, CI 0.96–1). Permutation test in both models were *P* < 0.01 (Supplementary Fig. S3).

### Urine bile acid and steroid concentrations of ADG-groups

**Figure 5:**
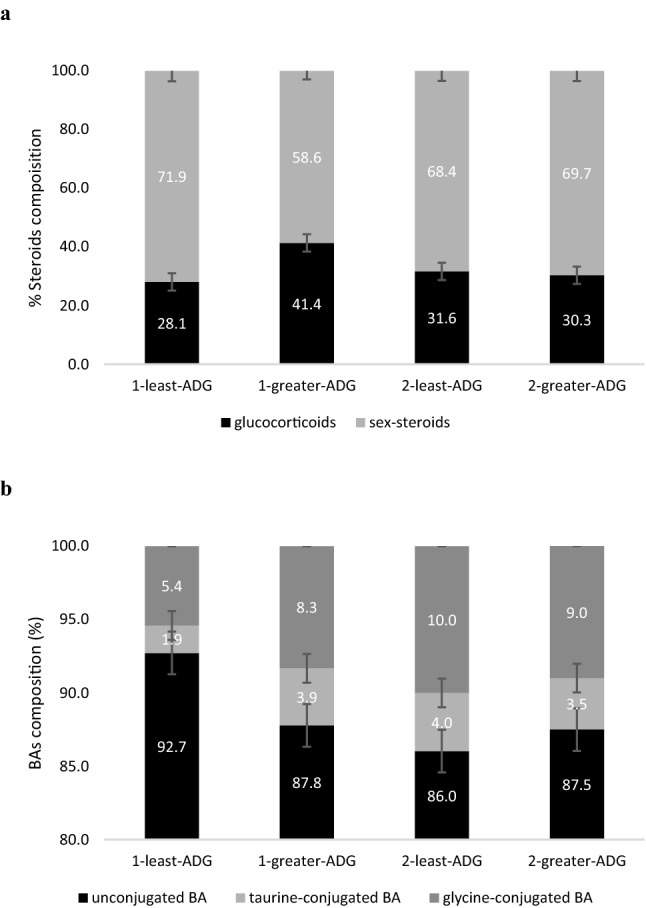
Urine steroids (**a**) and bile acids composition (**b**) (%) for ADG-classification in stage-1 and stage-2. Results are shown as means ± SE of all steers averaging the results of the 6 sampling times (n = 145). Urine glucocorticoids and sex-steroids (testosterone + progesterone) tended to be significant (*P* = 0.10) between ADG-classification in stage-1 but were not significant in stage-2. Urine bile acids composition of unconjugated, taurine-conjugated and glycine-conjugated were different (*P* < 0.05) in stage-1 but did not differ in stage-2.

Most bile acid (BA) and all detected steroid concentrations were affected by time of sampling (*P* < 0.05). Sampling time was again identified to be significantly and independently associated with ADG-groups (Supplementary Table S7). Of the total BAs in urine, unconjugated BA (%) were higher in least-ADG groups as compared to greater-ADG in stage-1, and higher than both ADG-phenotypes at stage-2 (Fig. 5). As a percent of total detected steroids, glucocorticoids tended to be lower (*P* = 0.10) and sex-steroids to be higher in least-ADG compare to greater-ADG in stage-1 but were not different in stage-2.

**Figure 6:**
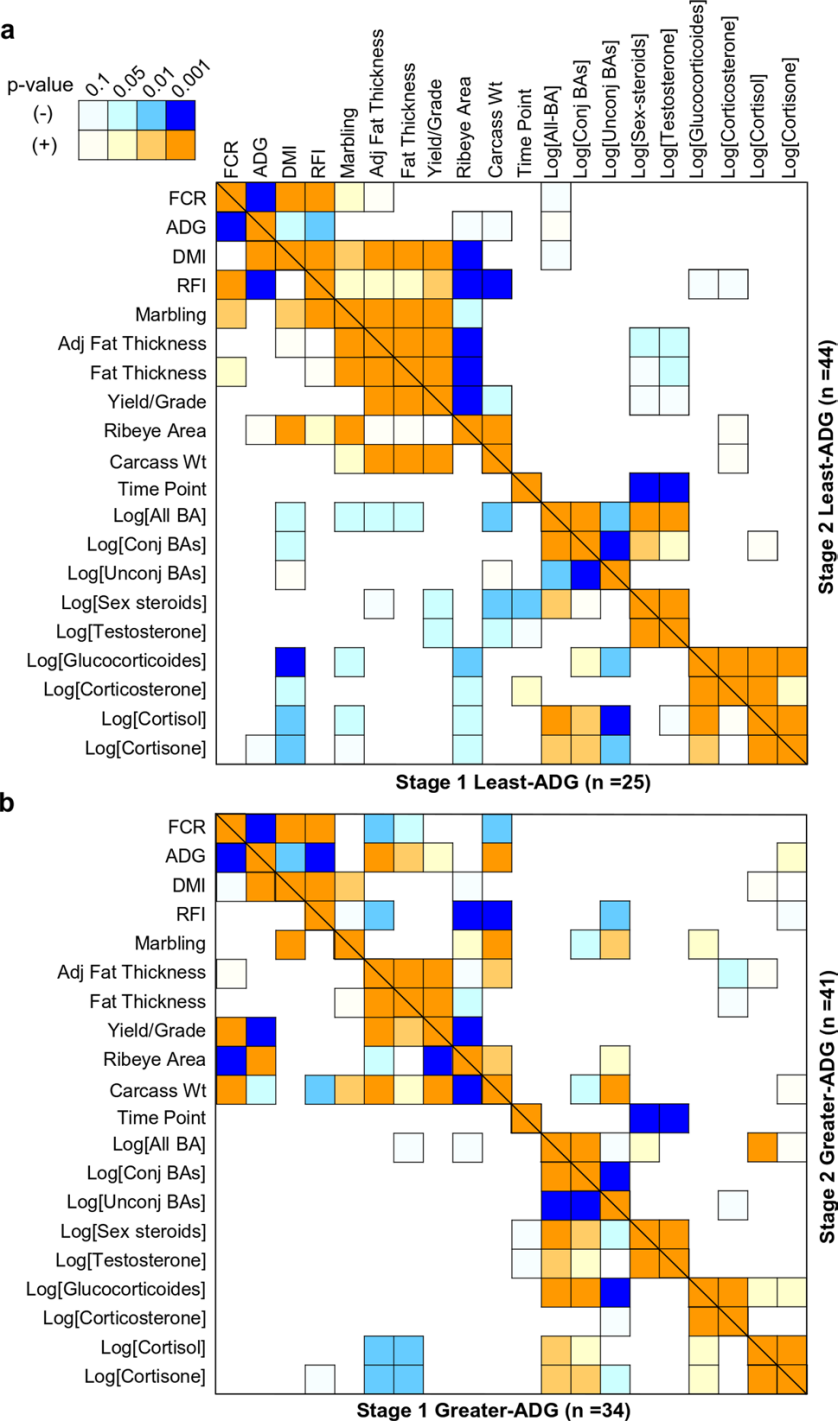
Pearson correlations (r) of bile acids and steroids with productive traits for least (**a**), and greater (**b**) ADG-classification in both stages.

There were distinctive associations of BA and steroids with performance and carcass quality across ADG-classification depending on stage (Fig. 6). In both stages, the feed conversion ratio (FCR; i.e. the DMI/ADG) FCR was positively associated with marbling (r > 0.34; *P* = 0.05) in least-ADG-groups while FCR was negatively associated with adjusted-fat-thickness (r > − 0.31; *P* = 0.05) in greater-ADG-groups. In stage-2, the concentration of unconjugated-BA was associated with marbling (r = 0.48; *P* < 0.01), ribeye-area (r = 0.36; *P* < 0.01), and carcass-weight (r = 0.53; *P* < 0.001) in greater-ADG but were not associated in least-ADG-group. The sex-steroids (sum of the concentration of testosterone and progesterone) were negatively associated (*P* < 0.01) with carcass traits in least-ADG-groups in both stages. Glucocorticoids (sum of the concentration of cortisol, cortisone and corticosterone) were associated with marbling differently in stage-1 in least-ADG (r = − 0.45; *P* = 0.01), and stage-2 in greater-ADG-group (r = 0.34; *P* = 0.05).

A predictive equation for least/greater-ADG was developed using a stepwise nominal logistic regression using log transformed urine bile acid and steroid concentrations (R^2^ = 0.61; *P* < 0.01; AUC = 0.97). The standard errors of the estimates are in supplementary table S8. Cutoff values of bile acids and steroids to predict least/greater-ADG are in Supplementary Table S9. The accuracy of ADG prediction by urine bile acids and steroids was 94% (91.2% for greater-ADG and 96.7% for least-ADG).

$$= 46.40 + 5.95 \times Log \left[Non-conjugated\right]+1.17 \times Log\left[DCA\right]-6.02 \times Log\left[GCDCA\right]+ 7.39 \times Log\left[TCDCA\right]-1.89 \times Log\left[\omega -MCA\right]-2.63 \times Log\left[CA\right]-5.77 \times Log\left[DCA\right]+ 5.76 \; Log[corticosterone] + 4.53\; Log[Cortisol] + 1.02 \times Log[Cortisone] + 13.53 \times Log[GCA] + 12.95 \times Log[GDCA] + 1.16 \times Log[GLCA] -13.76 \times Log[glucocorticoides] -9.19 \times Log[glycine-conjBA] + 1.25 \times Log[GHDCA] -1.01 \times Log[LCA]+1.02 \times Log[Progesterone] -5.68 \times Log[sex-steroids] + 6.85 \times Log[sum \; of \; all \; Steroids] \pm 2.45 \times Log[taurine-conjBA]) -1.46 \times Log[TDCA)]+ 0.70 \times Log[Testosterone]+5.30 \times Log[T-\alpha -MCA] -0.32 \times Log[UDCA] -3.11 \times Log[\alpha -MCA].$$

## Discussion

Improving production efficiency while obtaining quality meat is an increasing agricultural need from farm-to-table. Conventional livestock performance selection relies on the recording of feed intake over time in high-cost special facilities to identify efficient phenotypes, considered a moderately heritable^15,16^. More recently, animal breeding has evolved from conventional to molecular breeding^17^. While marker-assisted selection using genomics has become a reliable mainstream practice, the SNPs and GWAS do not fully explain the molecular basis of phenotypic variations in complex traits^18^. In this study, we have used urinary metabolomics to identify novel molecular biomarkers and biochemical pathways with the potential to improve cattle selection for feed efficiency and carcass quality.

The ADG-phenotypic expression represents one of the metrics to evaluate feed efficiency. The inter-animal ADG -variation stems from the interaction of many metabolic processes influenced, in turn, by physiological status and herd management^19^. In our study, extreme ADG phenotypes were selected independently from DMI in two different diets in growing animals. The selection criteria in both stages (diets) were the same. Steers selected in stage-1 for greater ADG had higher yield grades at slaughter. In stage-2 carcass traits were not different between selected ADG-groups likely due to an increase in the growth rate of the smaller animals promoted by high-energy diets^20^. The association of feed efficiency with carcass traits was previously evaluated based on RFI selection on growing beef cattle on a high-concentrate diet^21–23^. While the outcomes may differ across different metrics for feed efficiency; selection for RFI resulted in slight negative impacts on growth and reduce carcass quality^21^ and others, in contrast observed a reduction of the proportion of visceral organs which impact energy expenditure and hence protein turnover with a greater muscle content in the carcass^22^. The evidence in the scientific literature is certainly variable in the breed, sex and stage of physiological maturity of the cattle employed. This emphasizes the relevance of understanding the biological basis of animal traits to better define sustained progress on selection for feed efficiency and its consequences on carcass traits of beef cattle.

In our study, a distinctive urine metabolic fingerprint of the ADG-phenotypic groups was identified using non-targeted metabolomics and multivariate analysis, suggesting metabolic differences between ADG-phenotypical selection similarly to previous results in metabolomics analysis in rumen^13^ and multiple-tissues in beef-cattle^14^. These previous differences were involved in the metabolism of alpha-linolenic acid, unsaturated fatty acids, glycerophospholipids, taurine, and hypotaurine, creatinine, cysteine and methionine as well as bile acids and steroids, which were all in common with urinary factors characterized here. We identified for the first time metabolomic differences in sampling-time, diet, and breed type but only diet and breed type were both related to ADG-metabolome differences. Breed type had an effect in almost half of the untargeted metabolites identified for selected ADG-groups and only MARC-3xCharolais metabolome were not in least-ADG in both stages. Prior metabolomics studies reported differential molecular mechanisms of breeds to adapt to the environment^9^ and diet^24^, and association between feeding system and breed on fatty acids composition in beef muscle^25^. Selection for ADG and carcass quality is a complex trait of metabolic diverse expression in common across different tissues and biofluids, and some of these metabolic mechanisms may excel by breed and environment.

Using biomarkers analysis, we identified a small number of urine untargeted BA and steroid metabolites with excellent sensitivity and specificity for ADG-classification distinctive for each diet. Then, a predictive equation for ADG-(least/greater)-independently of diet and time sampling- was develop with a > 90% of accuracy using urine bile acid and steroid concentrations selected by stepwise analysis. A potential screening tool of feed efficiency that could be implement after weaning. Nevertheless, this algorithm requires further validations, including a larger cohort of animals, to warrants the accuracy of suggested cutoff values.

Urine steroids and BA are cholesterol-derived metabolites involved in the regulation of different biological pathways mainly in metabolic homeostasis and maintenance^26^. Total BA concentration is traditionally used as a clinical index of liver pathology in cattle^27^, although the advance of current quantification techniques of BA and steroid profiling makes it possible to elucidate their roles in diet intervention^28^, breeding^29^ and metabolic functions^30^. Here a total 18 BAs and 5 steroids were detected in cattle urine, with conjugated-BAs positively associated with sex-steroids and glucocorticoids in both diets. Previously, similar associations of urine bile acid and glucocorticoid concentration were shown to be related to the level of dietary cholesterol in monogastric (humans)^31^. In ruminants, cholesterol is exclusively obtained by hepatic de novo synthesis^32^, which is associated with cattle efficiency by reductions in both in gene expression of hepatic lipid synthesis^33^ and ultimately cholesterol concentrations^14,34,35^. In our results, the urine concentration of conjugated-BAs was higher in a concentrate-diet while differences by ADG-groups in conjugated-BA were only observed in a forage-based diet. In high-grain diets, conjugated-BA were increased by signature microbial population changes in the small intestine of Angus-cattle^28^. Differences in urine conjugated-BA concentrations in relation to cattle efficiency may reflect the hepatic capacity of cholesterol synthesis, transport, and oxidation, which in turn depends on the diet. An increased conjugated-BA, furthermore, triggers metabolic activities of microbes (e.g. glycerophospholipids) in the lower GI tract of beef cattle, and is suggested to be one of the links of increased unhealthy fatty acids in beef resulting from high-corn diets^28^. Thus, the association of urine BA concentrations with carcass quality may reflect differences in nutrient absorption at the small intestine and/or changes in the intestinal microbiome.

The endogenous concentration of sex-steroids and glucocorticoids were previously suggested as a useful tool for the prediction of beef carcass quality^36,37^. Endogenous sex-hormones exhibit anabolic effects. For instance, testosterone regulates body composition by increasing skeletal muscle mass while decreasing fat mass^38^. Similar to our results, carcass leanness was associated with testosterone in growing beef bulls^36,39^. The relationship between testosterone concentration and cattle performance-within the same animal category-has not been extensively reported. In twenty “Red Danish” bulls, plasma testosterone measured 3-times from 4 to 10 months of age was not associated with the rate of gain or linear growth^40^. Nevertheless, glucocorticoid concentrations were related to both productive performance and carcass quality in our results, and the direction of their correlation depended on ADG-group and diet. Plasma cortisol and fecal corticosterone previously measured in beef cattle on a high-corn diet during the finishing-period were indicators of production traits in a contradictory manner between studies^41–43^. Glucocorticoids are steroids produced by the adrenal cortex and play a key role in energy homeostasis by peripheral tissues including skeletal muscle^44^. Physiologically concentration of endogenous glucocorticoids has major stimulatory effects on food intake and energy balance, and is well understood in monogastric models^45^, which are a major adaptive regulatory mechanism for phenotype and environment.

Based on our data, urine metabolomics analysis offers metabolic phenotypes that are both quantitative and highly targeted biomarkers for feed-efficient animals for growth on forage- and concentrate-based diets. Noninvasive, low-cost, and minimal-training time of urine sampling compared to other biofluids makes it an especially attractive screening tool for performance and carcass quality that could potentially be included in breeding programs to refine phenotypes and improve the accuracy of predictions of economically important traits. Although high-throughput metabolomics approaches for biomarker discovery at the molecular level are available, challenges remain to translate these technologies as affordable analytical techniques coupled to transparent and translatable data mining to farm programs. Target analysis of urinary bile acid and steroid concentrations may represent a potential screening tool to implement in breeding programs.

## Experimental procedures

### Study design and samples details

Research protocols were approved and monitored by the USDA, ARS, U.S. Meat Animal Research Center Institutional and Animal Care Committee in accordance with the Guide for the Care and Use of Agricultural Animals in Agricultural Research and Teaching (2010). All authors complied with the ARRIVE guidelines. Calves (n = 75) used in this experiment were the steer progeny of Red Angus, Charolais, and Simmental sires bred by artificial insemination to four dam breeds: MARC II Composite (¼ Hereford, ¼ Angus, ¼ Gelbvieh, ¼ Simmental), MARC III Composite (¼ Hereford, ¼ Angus, ¼ Pinzgauer, ¼ Red Poll), ½ MARC II X ½ MARC III, and ½ MARC II X (¼Red Angus X ¼ Angus).

#### Stage-1 (forage based-diet)

After weaning, steers received an implant containing 80 mg of trenbolone acetate and 16 mg estradiol (Revalor-IS; Merck), and were housed in a facility with Calan Broadbent electronic headgates (American Calan, Inc.,Northwood, NH) to measure individual feed intake. Steers were trained to use Calan Headgates during an adaptation period (approximately 21 days).

At the beginning of the study (0 day), study population mean age and weight of cattle (± standard deviation) was 271.4 ± 5.0 days and 301.7 ± 31.9 kg, respectively. The diet consisted of 30.0% chopped alfalfa, 69.8% corn silage, and 0.2% salt on a DM basis.

Feed intake was measured for 84 days with cattle weighed on day 0, 1, 21, 42, 63, 83 and 84 of the experiment. Daily feed offered was recorded and orts determined weekly. A urine sample was collected at day 0, 42, and 83 during the scheduled weigh dates.

#### Stage-2 (concentrate based-diet)

Following the completion of stage 1, steers were transitioned to a concentrate-based ration over a 21-day period and remained on that diet for the remainder of stage 2. On a DM basis, the diet consisted of 8.00% chopped alfalfa, 67.75% rolled corn, 20.00% wet distillers grain with solubles, and 4.25% vitamin/mineral supplement with Rumensin (Elanco, Greenfild, IN). Forty-two days after stage 1 steers received an implant containing 120 mg of trenbolone acetate and 24 mg of estradiol (Revalor-S, Merck Animal Health, Madison, NJ) and feed intake and body weight gain data were collected for 84 days. Body weight measurements were determined at 0, 1, 21, 42, 63, 83 and 84 days on study. Daily feed offered were recorded and orts determined weekly. At the beginning of stage-2 (0 day), mean age and weight of cattle (± standard deviation) was 397.3 ± 5.0 days and 448.6 ± 41.8 kg, respectively. A urine sample was collected at day 0, 42, and 83 during the scheduled weigh dates.

#### Urine collection

On days 0, 42, and 83 for each stage-1/2 urine samples (~ 50 mL) were collected by preputial stimulation and immediately centrifuge 10,000*g* for 10 min to remove particulates (4 °C) before storage at − 80 °C. No urine sample could be collected by stimulation 23% (n = 19) of the selected steers in stage-1 and 11% (n = 9) of the selected steers in stage-2.

### Steer selection by divergent growth for each stage

**Figure 7:**
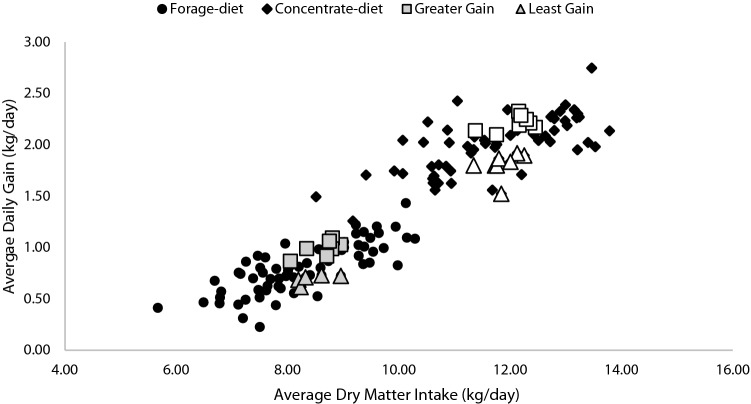
Urine sampling selection. The animals groups were selected by the greatest average daily gain (greater-ADG; grey-square; n = 7) and the least ADG steers (least-ADG; grey-triangle; n = 5) with similar average dry matter intake in stage-1 (black-circles) and the greatest average daily gain (greater-ADG; white-square; n = 8) and the least ADG steers (least-ADG; white-triangle; n = 8) with similar average dry matter intake in stage-2 (black-diamonds) from the total population (n = 75) evaluated. Steers classified as greater- or least-ADG in stage-1 were not the same steers classified in stage-2.

Steers were selected according to differences in ADG at each stage with those with the greatest and least ADG, whose dry matter intake was within 0.32 SD of the mean intake (Fig. 7). After the end of intake study (stage-2), selected steers in both stages received the same ration ad libitum and remained in the same pen until slaughter (5–8 days) at an abattoir. Carcass data was collected using the VBG2000 beef carcass grading camera (Vision For You, L.L.C., Dakota Dunes, SD).

### Sample preparation for untargeted metabolomics analysis

All urine sample extractions and LC–MS analysis were randomized across time sampling to avoid batch effects. Duplicate samples of each urine were extracted for metabolomics analysis within batches. Both extraction and chromatographic solvents were UPLC-MS Optima Grade (Fisher Chemical Ltd., Waltham, MA). Sample preparation was adapted from previous reports^46^. Briefly, 50 µL urine aliquots were diluted with 150 µL of water, vortexed and centrifuged at 16,000×*g* for 10 min at 4 °C. The supernatant was filtered at 0.22 µm by centrifugation in Spin-X LC (Fisher Chemical Ltd., Waltham, MA) membrane-tube filters (5000×*g* for 5 min at 4 °C) before transfer to LC-vials.

### UPLC-qTOF spectral acquisition of urine samples

The UPLC/MS analysis was performed using a Waters ACQUITY ultra-performance liquid-chromatography (UPLC) system (Waters Corporation, Milford, MA) equipped with an auto sampler and coupled with a hybrid triple quadrupole-time of-flight mass spectrometry (XEVO-G2-S-qTOF; Waters). Instrument calibration was performed before running the samples using 0.5 nM sodium formate. To obtain information regarding system suitability and stability, quality control (QC) samples were injected at regular intervals (every ten samples) throughout the analytical run. Quality control samples were prepared by mixing ~ 100 urine aliquots (10 µL), producing separate QC samples.

For analyses, samples were held at 4 °C, and analytes in 10 µL of urine extracts were separated on a 2.1 × 50 mm × 1.7 µm Acquity UPLC BEH C18 column (Waters Corp.) held at 40 ºC using a 0.4 mL/min flow of 0.1% formic acid in water (mobile phase A) and 0.1% formic acid in acetonitrile (mobile phase B) with the following gradient: 99% A from 0 to 2 min; 85% A at 4 min; 50% A at 8 min; 5% A at 12 min; 99% A at for 16 min followed by 2 min of re-equilibration. All gradient ramps were linear.

Mass spectrometry was performed in both positive and negative modes. The capillary voltage was 3.2 kV and 2.4 kV for positive and negative mode, respectively. The system parameters were set as follows: source of temperature 120 °C, desolvation temperature 350 °C, and cone gas flow (nitrogen) 25 L/h and desolvation gas flow (nitrogen) 900 L/h. Data were collected in a centroid mode using the lockspray to ensure accuracy and reproducibility. Leucine enkephalin was used as lock-mass in a 2 ng/mL concentration solution. The lockspray frequency was set at 15 s, and the lock mass data were average over 15 scans for correction. The scan mass range was from 50 to 1200 *m/z* using an extended dynamic range. The MS/MS analysis was carried out by ramping the collision energy from 10 to 50 V using argon as a collision gas.

### Untargeted-metabolomics data processing

Raw data obtained from UPLC-qTOF analysis was analyzed using Progenesis QI v1.0 software (Waters Corp.). The data was aligned, deconvoluted, and normalized using total ion intensity. Solvent blanks were run between samples and each mass was checked against the blank run to exclude possible sources of contaminations. The coefficient of variation (CV) of the mass abundance for all biological replicates, was calculated across pooled repeated samples for each feature and those with a CV above a 20% cutoff were removed. Metabolites were identified by comparison with online Bovine Metabolome Database (http://www.cowmetdb.ca/*)* using exact *m/z* values and retention times. The identities of selected metabolites were confirmed by MS/MS fragment ion analysis using Mass-Fragment application manager software (Water MassLynk v4.1, Waters Corp.). The MS/MS fragmentation of the molecules was compared, with ChemSpider database (www.chemspider. com), by way of chemically intelligent peak-matching algorithms.

### Urine bile acid and steroid quantification

Endogenous urinary steroids and bile acids were quantified using isotopic enrichment and authentic calibration standards detected by LC–MS/MS with electrospray ionization and multiple reaction monitoring (MRM) on an API 6500QTRAP (Sciex, Framingham, MA, USA) as previously reported for plasma^47^. Urine extraction was performed by solid-phase extraction (SPE) using Oasis-HLB SPE 96-wellplates 30 mg (Milford, MA, USA). Briefly, 100 µL urine aliquots were spiked with 10 µL of methanol containing a suite of deuterated bile acids and steroids at 12.5 nM, vortexed, and loaded onto SPE cartridges pre-conditioned with 1 mL MeOH, followed by 1 mL H_2_O. Loaded cartridges were then washed with 1 mL H_2_O and eluted with 2 mL MeOH. The eluate was evaporated under vacuum at room temperature and reconstituted in 100 µL of 50:50 MeOH:H_2_O (v/v). Extracts were separated on a 2.1 × 100 mm, 1.7 μm Acquity C18 BEH column using a Shimadzu Nexera X2 UPLC (Shimadzu, Kyoto, Japan). Analytes were quantified using internal standard methods with surrogate/analyte associations for response ratio calculations. Data was processed with AB Sciex MultiQuant v 3.0.1.

### Statistical and machine learning data analysis

#### Animal traits

The feed conversion ratio (FCR) was calculated as the quotient of DMI divided by ADG. The residual feed intake (RFI) was calculated as the residual obtained from the linear regression of DMI on ADG and calculated metabolic body weight at the midpoint of each stage. Feed intake, body weight traits, FCR, RFI, age, and carcass traits were analyzed within stage using the mixed procedure of SAS (Cary, NC). The model included ADG classification as a fixed effect and animal nested within classification as a random effect with the denominator degrees of freedom set to the Kenward-Roger method.

#### Untargeted urine metabolomics analysis

The resulting data matrix was imported into MetaboAnalyst version 5.0^48^ for subsequent univariate/multivariate analysis. To account for repeated measures structure of the data, the focus of our methodology for analysis was to explain the metabolic changes across steers over time and stage. ANOVA-simultaneous component analysis (ASCA) was conducted to identify the effect of time or stage (diet) on the metabolome urine profile of an ADG-classification (least-ADG vs. greater-ADG steers) using a significance level of *P* < 0.05. Permutation testing (1000 times) was performed to minimize the possibility that observed effects were by chance. Leverage was used to evaluate the importance of the metabolite to the model, and square prediction error (SPE) was used as a test of the fitness of the model for the particular metabolite. Variables with low SPE and higher leverage had a significant contribution to the model and were selected as influentially affected compounds. Two-way ANOVA univariate analysis was conducted to evaluate the effect of breed type on metabolomic profiles of selected ADG-groups. Post hoc analysis with false discovery rate (FDR) correction was performed on both main effects level and interaction of the metabolites identified. Hierarchical cluster analyses were used to group metabolite by ADG-classification time-dependent changes (FDR < 0.05). The interactive principal components analysis (iPCA) was conducted in both two-way-ANOVO and ASCA to identify and visualize stage and breed type effects of least-ADG vs. greater-ADG steers. Pathway analysis was performed using a *Bos taurus* pathway library, which integrate global pathway enrichment analysis and relative between centrality pathway topology analysis from MetaboAnalyst 3.0 software. The identification and visualization of the top altered pathways were based on KEGG (http://www.genome.jp/kegg/) database sources^49^. Receiver operating characteristic curve (ROC) analysis was performed to evaluate the minimum number of untargeted metabolites that have the sensitivity and specificity to distinguish between ADG-groups using a linear support vector machine (SVM) algorithm. These models were assessed by area under the curve (AUC) and prediction accuracies by cross-validation. The discriminating ability of AUC were considered as follow: 0.9–1.0 = excellent; 0.8–0.9 = good; 0.7–0.8 = fair. A ROC curve plot the true positive rate (Sensitivity) as a function of the false positive rate (100-Specificity) for cut-off points of selected metabolites calculated with a 95% confidence interval. The significance of the model was further validated using permutation test.

#### Targeted urine bile acids and steroids

BA and steroid concentrations were log-transformed to achieve normality. The generalized linear mixed model (GLIMMIX) procedure of SAS (SAS Institute, Cary, North Carolina) were used to examine the bile acid and steroid responses to experimental variables. The model included time, stage, breed type, ADG-classification and interactions as fixed effect, residual of steers nested by stage as a random effect, with time as the repeated measure, and spatial power covariance as structure of sampling interval.

Pearson’s correlations were performed to identify the associations between urine bile acids and steroid concentrations with performance and carcass traits. Correlation strength was considered strong (|r|≥ 0.7), moderate (0.7 >|r|≥ 0.4) or weak (0.4 >|r|≥ 0.1) (15). A predictive equation for ADG-classification using bile acids and steroids log transform concentrations was performed by stepwise nominal logistic regression analysis with a JMP Pro version 15.0 (SAS Institute, Cary, North Carolina). Basically, for each ADG-category, multiple regression is run to predict the odds ratio into that category. The odds ratios are then converted into probabilities, and the most likely class is chosen. For ADG-prediction model building, data were divided into a 75/25 training:validation set splits to avoid over-fitting and minimize model reliance on sample selection. Validation set was stratified by ADG-classification to balance training set representation and used to test model fit probabilities. The model sensitivity (true positive), specificity (true negative) and accuracy (proportion of true results, either true positive or true negative) was evaluated from confusion matrix based on the final model training set randomly chosen.

**Figure 8:**
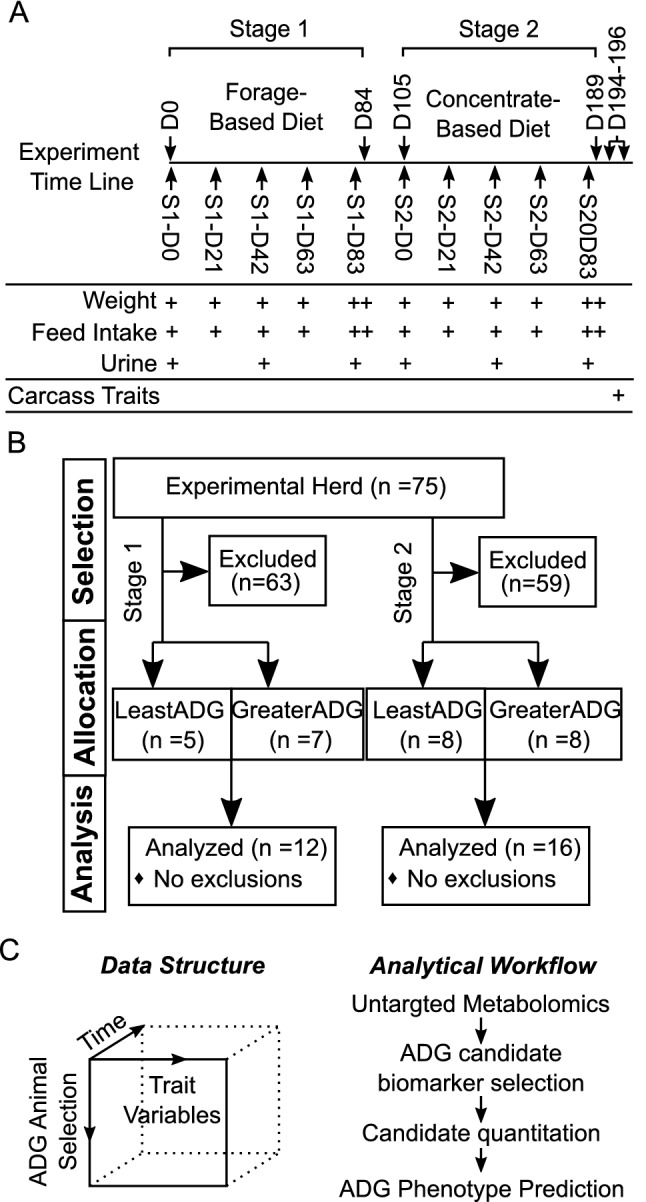
Graphical summary of study design. MARC Nutritional feeding study (**A**) time line and animal traits measured (**B**) selection of animals for greater and least average daily gain with similar average dry matter intake in stage-1 and stage-2 (**C**) characteristics along with the data and data analysis workflow covered in the current study.

An overview of the experimental design and data analysis is presented in the Fig. 8.

## Supplementary Information

Supplementary Figures.

Supplementary Tables.

## Data Availability

All data generated or analyzed during this study are included in this published article and its Supplementary files.
