# Supplementary material for: Non-invasive metabolomics biomarkers of production efficiency and beef carcass quality traits
Source: Sci Rep. 2022 Jan 7;12:231. doi: 10.1038/s41598-021-04049-2 (PMC8742028; doi:10.1038/s41598-021-04049-2)
Supplement: Supplementary file 1 — Supplementary Figures. [file 41598_2021_4049_MOESM1_ESM.docx]

**Supplementary Figure 1**. (a) Metabolites distribution before and after normalization. (b) The scree plots displaying the relationship between eigenvalues and factors. (c) ASCA score plots for factor time ADG-phenotypes, and their interactions. (d) Model validations through permutations, as demonstrated by significance levels of p < 0.01 for the ADG-phenotype, time, and non-significant interaction.

**
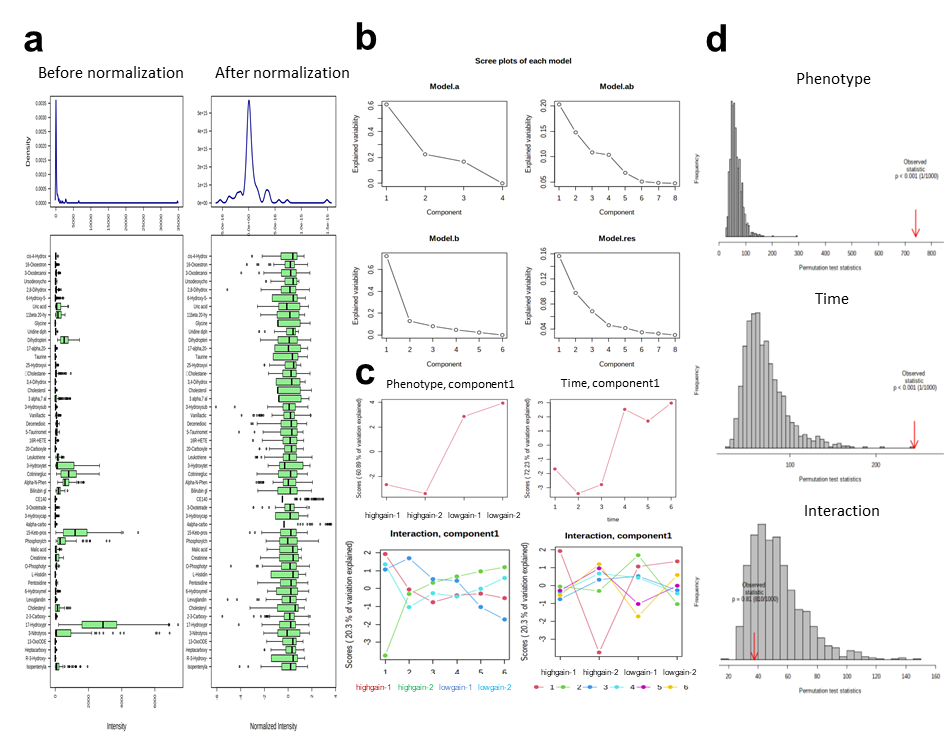
**

**Supplementary Figure 2.** Heatmap of urine untargeted metabolites for breed type effects **
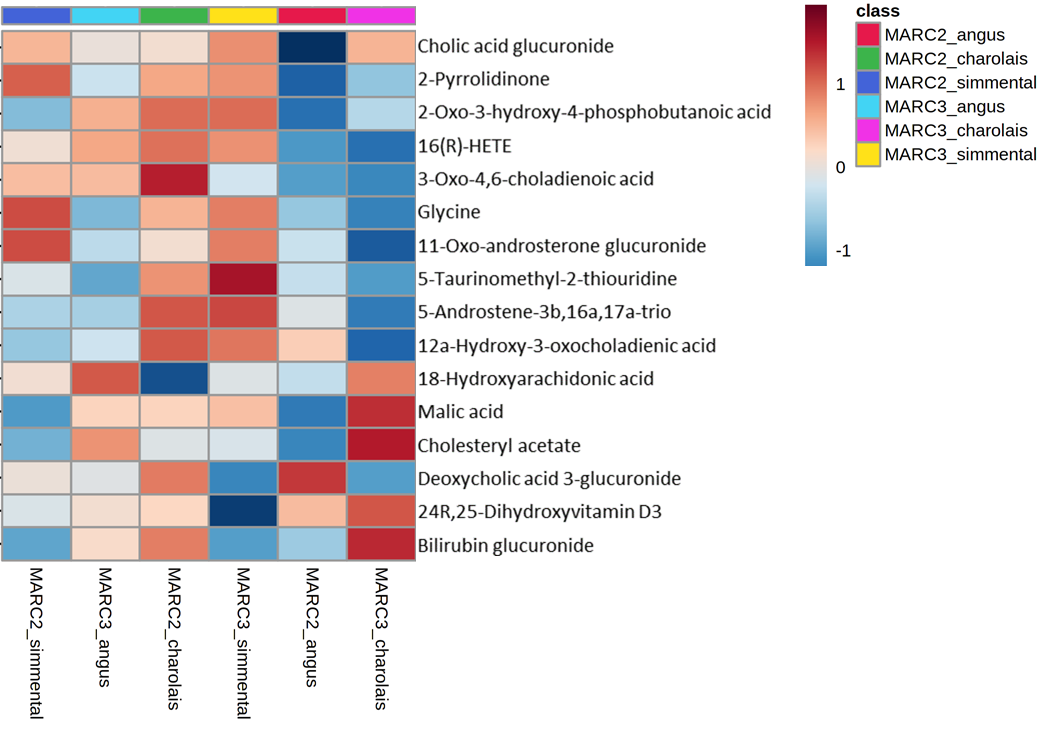
**

**Supplementary Figure 3**. Result of the ROC analysis of the untargeted metabolites, by androsterone sulfate, 11-hydroxyprogesterone-glucuronide, deoxycholic acid glycine conjugated and taurochenodeoxycholate in stage-1 (AUC=0.93, CI:0.84-0.99) while dexocholic acid glycine conjugated, progesterone and taurochenodeoxycholate were identified in stage-2 (AUC=0.99, CI:0.96-1). A) ROC curve plot b) The average accuracy based on 100 cross validations c) confusion matrix d) Permutation test in both models were P <0.01.

Stage-1

| a  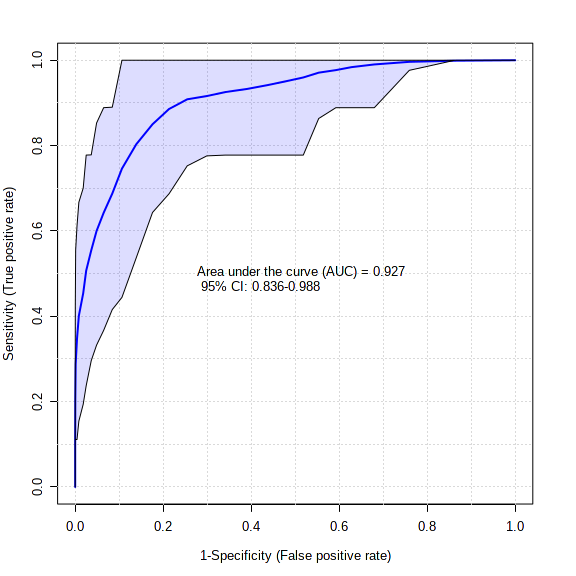 | b  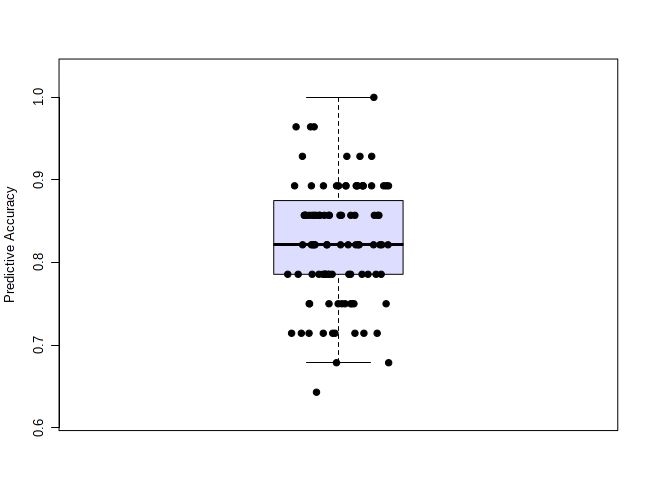 |
| --- | --- |
| c  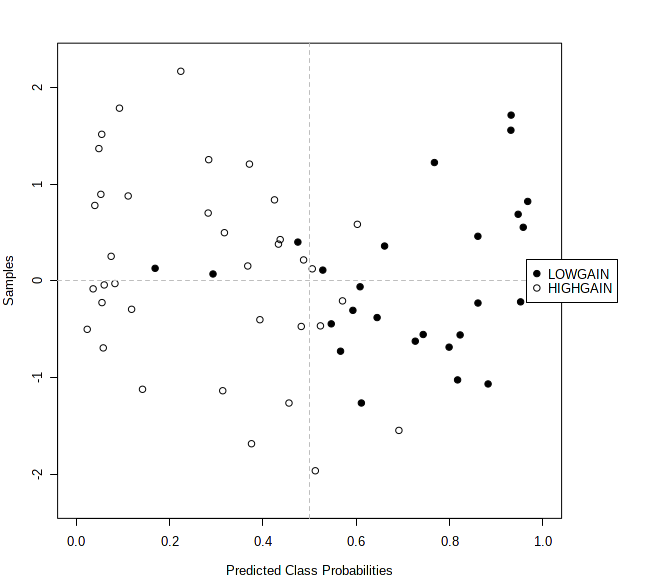 | d  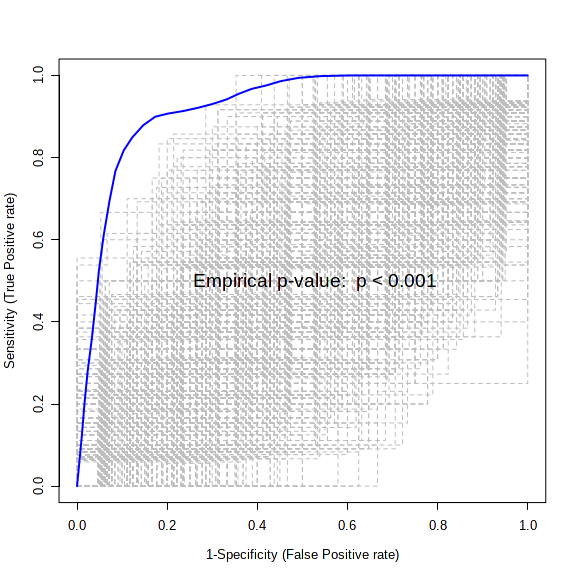 |

Stage-2

| a  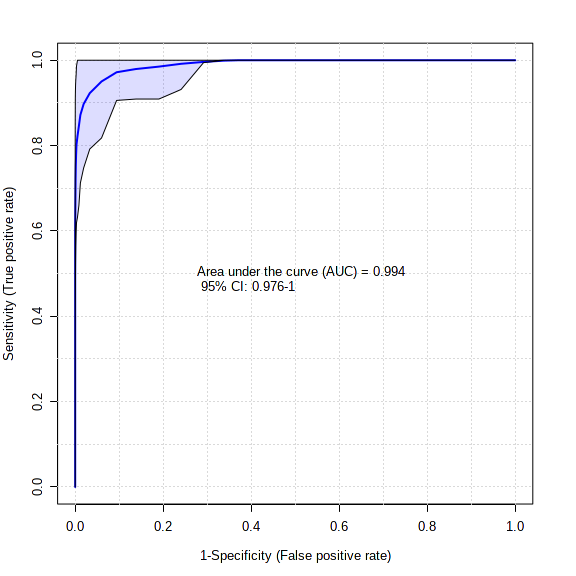 | b  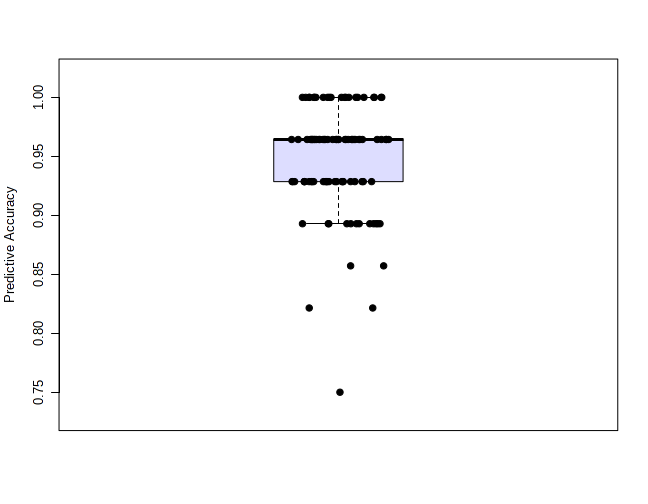 |
| --- | --- |
| c  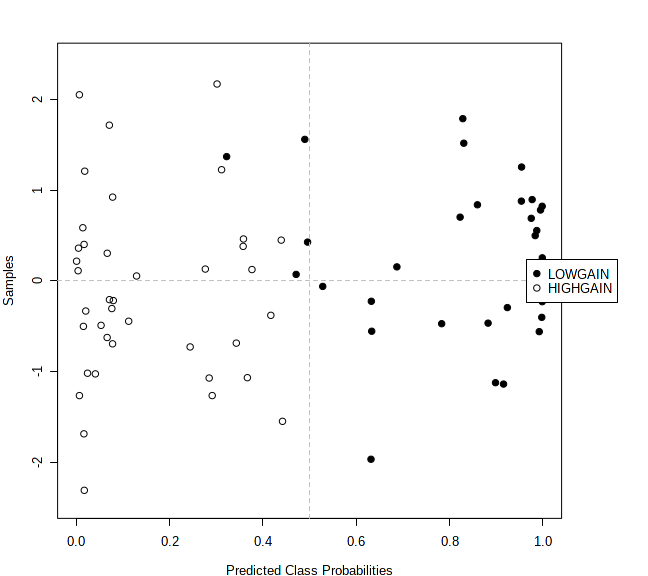 | d  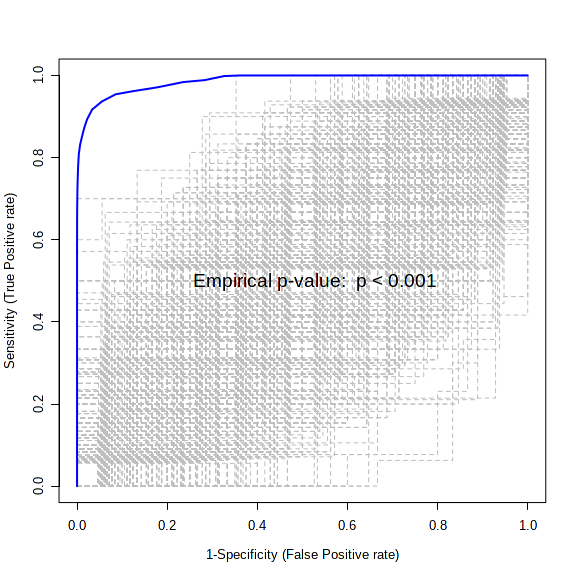 |
